# Tools to Image Germplasm Dynamics During Early Zebrafish Development

**DOI:** 10.3389/fcell.2021.712503

**Published:** 2021-08-13

**Authors:** Andreas Zaucker, Claire A. Mitchell, Helena L. E. Coker, Karuna Sampath

**Affiliations:** Warwick Medical School, University of Warwick, Coventry, United Kingdom

**Keywords:** germ plasm, zebrafish, dynamics, tools, mounting, germ granules, germ cells, imaging

## Abstract

During the first day of zebrafish development, ribonucleoprotein (RNP) complexes called germplasm form large aggregates that initially segregate asymmetrically during cleavage stages. After zygotic genome activation, the granules break into smaller fragments that associate with the nuclear membrane as perinuclear (germ) granules toward the end of gastrulation. The mechanisms underlying the highly dynamic behavior of germ granules are not well studied but thought to be facilitated by the cytoskeleton. Here, we present efficient mounting strategies using 3d-printed tools that generate wells on agarose-coated sample holders to allow high-resolution imaging of multiplexed embryos that are less than one day post-fertilization (dpf) on inverted (spinning disk confocal) as well as upright (lattice light-sheet and diSPIM) microscopes. In particular, our tools and methodology allow water dipping lenses to have direct access to mounted embryos, with no obstructions to the light path (e.g., through low melting agarose or methyl cellulose). Moreover, the multiplexed tight arrays of wells generated by our tools facilitate efficient mounting of early embryos (including cleavage stages) for live imaging. These methods and tools, together with new transgenic reporter lines, can facilitate the study of germ granule dynamics throughout their lifetime in detail, at high resolution and throughput, using live imaging technologies.

## Introduction

The germline is essential for reproduction and maintenance of species. The zebrafish germline is founded by a small number of cells, the primordial germ cells (PGCs), that receive a specialized cytoplasmic substance called the “germplasm” (Dosch, 2015; Marlow, 2015). Germplasm forms by the aggregation of granules that are composed of Ribonucleoprotein (RNP) complexes specific to the germline, hereafter called “germ granules” (Voronina et al., 2011; Kaufman et al., 2018; Eno et al., 2019). The number of PGCs in early larval zebrafish correlates with sex development, with low numbers predominantly developing as males and high numbers developing as females (Tzung et al., 2015). Intricate molecular mechanisms for the localization, aggregation and segregation of germplasm are thought to ensure that the number of PGCs specified is in the appropriate range to facilitate development of both sexes.

During the first cleavage divisions, germ granules aggregate in the distal corners of the cleavage furrows. This leads to the formation of four masses of germplasm at the four-cell stage. After cellularization, the cells that receive the masses become PGCs. Initially, germplasm is only inherited by one of the daughter cells during cleavage and early blastula stage cell divisions. Around the 1k-cell stage, the masses begin to fragment before gradually releasing their granules into the cytoplasm during the transition of oblong into sphere stage (Eno et al., 2019).

Work by the Pelegri lab and others laid the foundation for understanding the processes controlling germplasm behavior, especially during the first cleavage divisions (Moravec and Pelegri, 2020). Germplasm segregation is thought to be facilitated by the cytoskeleton and motor proteins, and germ granules have been shown to associate with both microtubules and actin (Knaut et al., 2000; Strasser et al., 2008). Some cytoskeleton-associated factors and pathways regulating the cytoskeleton have been implicated in the process (Nair et al., 2013; Eno and Pelegri, 2018; Eno et al., 2018). However, later processes such as the uptake of germplasm into cells at the 16–32 cell stage, the initial asymmetric segregation, germplasm fragmentation and subsequent dispersal are less well understood. Moreover, much of what we know about germplasm has been inferred from *in situ* hybridization using fixed embryos, which does not capture the dynamic behavior of germ granules. These gaps in our knowledge can be filled by live imaging combined with molecular genetics of the components.

Recent high resolution microscopy technologies such as lattice light-sheet microscopy now provide unprecedented access to the dynamic behavior of germplasm by offering high speed volume acquisition combined with low photo-bleaching and photo-toxicity (Liu et al., 2018). The high speed of acquisition in these imaging methodologies enables the determination of germ granule kinetic parameters, which can support studies on the underlying molecular mechanisms, to provide a comprehensive cell biological understanding of germplasm and germ cells. The low photo-bleaching and photo-toxicity of these methods allows long-term live imaging of germplasm over many hours and even days; and studying changes in germplasm behavior across scales during embryonic development.

## Design of Tools

A standard live-imaging experiment in zebrafish comprises the following steps: (1) fluorescent labeling by using transgenic lines or by microinjection of live reporters, (2) mounting of embryos for imaging, (3) imaging on a microscope, (4) image processing, and (5) image analysis. For a successful experiment with high quality data, each of these steps need to be optimized (Megason, 2009). One prerequisite for the generation of quantifiable imaging data is a mounting method that facilitates standardized imaging of multiple embryos in the same orientation, and across experiments.

The size and shape of zebrafish embryos changes dramatically during embryonic development (Kimmel et al., 1995). In addition, the mounting method might need adjustments when using different microscopes, accounting for specifics such as the direction of imaging, inverted versus upright microscopes, sample holders used with the microscopes, and the types of objectives used, e.g., water dipping versus oil immersion lenses. Therefore, mounting methods need to be tailored for the specific stages imaged and the microscopes used for imaging.

The most common mounting strategies for imaging of zebrafish embryos are:

1. Mounting in a drop of molten low melting agarose (LMA) which is allowed to solidify. While the LMA is cooling down, the embryo can by oriented in the desirable orientation, and remains stable after the LMA has solidified (Hirsinger and Steventon, 2017).
2. Mounting in a highly viscous solution of methyl cellulose (Renaud et al., 2011).
3. Multilayer mounting in a very low non-solidifying concentration of low melting agarose for mounting embryos in fluorinated ethylene propylene (FEP) tubes (Kaufmann et al., 2012).
4. Using molds to cast wells (pockets) on agarose coated dishes that can serve as sample holders for imaging. The embryos sit in a largely stable orientation within the wells (Gehrig et al., 2009).

Historically, the two most common mounting methods for imaging of zebrafish embryos are mounting in a solidifying drop of low melting agarose (strategy 1) or in a highly viscous solution of methyl cellulose (strategy 2). Both of these mounting media are not ideal for imaging over longer periods of time. At the commonly used concentrations, LMA can compromise embryonic morphogenesis and oxygen supply to the developing embryo. Mounting in methyl cellulose is not stable over long periods of time and can also restrict growth at high concentrations (Kaufmann et al., 2012).

To overcome these limitations, alternative methods have been recently introduced wherein embryos are mounted in glass tubes or in FEP tubes (strategy 3). Very low, non-solidifying concentrations of LMA can be used as the mounting medium for embryos inside FEP tubes. The low LMA concentrations allow normal embryonic morphology, but at a slower rate of development (Kaufmann et al., 2012).

The main advantage of strategy 4, where embryos are mounted in wells on agarose-coated dishes, is that the use of viscous mounting media can be completely avoided (Gehrig et al., 2009). Low melting agarose is still used in some protocols to stabilize the orientation of the embryo (Kleinhans and Lecaudey, 2019). The mounting wells are generated in a manner similar to troughs for the loading of DNA gels during cooling down of agarose gels (Megason, 2009). The design of the mold (the comb) is a negative of the shape of the wells. Two technologies are commonly used to make molds: milling and 3d printing (Donoughe et al., 2018). This strategy is versatile and has successfully been used for imaging of zebrafish embryos on a variety of microscopes and in different formats (scales), ranging from single embryos to 96 well plate format and even larger array formats (Westhoff et al., 2013; Alessandri et al., 2017).

We set out to develop 3d-printed tools that generate wells in agarose-coated imaging holders that position embryos for the imaging of germplasm. The design of the molds had to fulfil four **main criteria:**

1. **No requirement for viscous mounting media.**
2. **Direct access for water dipping lenses to the embryo.**
3. **Embryo rests directly on the cover slip in glass-bottom dishes used for imaging on inverted microscopes.**
4. **They should allow normal development of the embryo.**

Any object placed in the light path between the object of interest and the objectives, e.g., overlying tissue or a cover slip, will impair imaging quality via phenomena such as refraction, absorption, reflection, and scattering of light. This also holds for materials that are thought to have the same refractive index as water, such as LMA. Accordingly, the use of LMA has been identified as a factor that reduces the quality of image acquisition on lattice light-sheet microscopes (Heddleston, 2019). Therefore, we designed our tools to facilitate mounting of embryos in a manner that avoids any additional materials in the light path between embryo and objectives, aside of the imaging medium **(criterium 1)**.

For imaging on upright microscopes equipped with water dipping lenses, our tools allow direct access of the excitation and imaging objectives to the embryo (**criterium 2**). For inverted microscopes, direct access of the objectives to the embryos cannot be achieved because the use of a cover slip cannot be avoided. However, our tools generate wells directly on the cover slip of a glass-bottom dish, with no agarose between the mounted embryos and the cover slip (**criterium 3**). The purpose of the wells is to hold the embryos in a stable position without overtly exerting pressure that could potentially interfere with normal development (**criterium 4**). This can be achieved by careful design of the mold according to the dimensions of the embryos at the stage to be imaged.

Our designs are for imaging embryos during the first day of development, with focus on stages when the embryo is roughly spherical, and prior to somitogenesis. Germplasm dynamics has not been well studied at these stages. Designing an exact negative of the embryo, e.g., a pin of exactly the same diameter as the embryo, would not achieve that because the embryo goes through changes in shape during development, and furthermore, embryos from different clutches can differ by tens to hundreds of microns in diameter (Wuhr et al., 2011).

A proven design to hold embryos in a specific position are conical designs, such as wedges, because the embryo slides into a position where it is held by the tapered walls of the well (Westhoff et al., 2013). Other often-used designs are cylindrical or half spherical structures to hold the yolk ball of the embryo for imaging lateral views (Wittbrodt et al., 2014; Campinho et al., 2018; Kleinhans and Lecaudey, 2019). However, for early embryos, the design needs to be optimized to exert only minimal pressure, that does not perturb normal development, for instance, during gastrulation.

**FIGURE 1:**
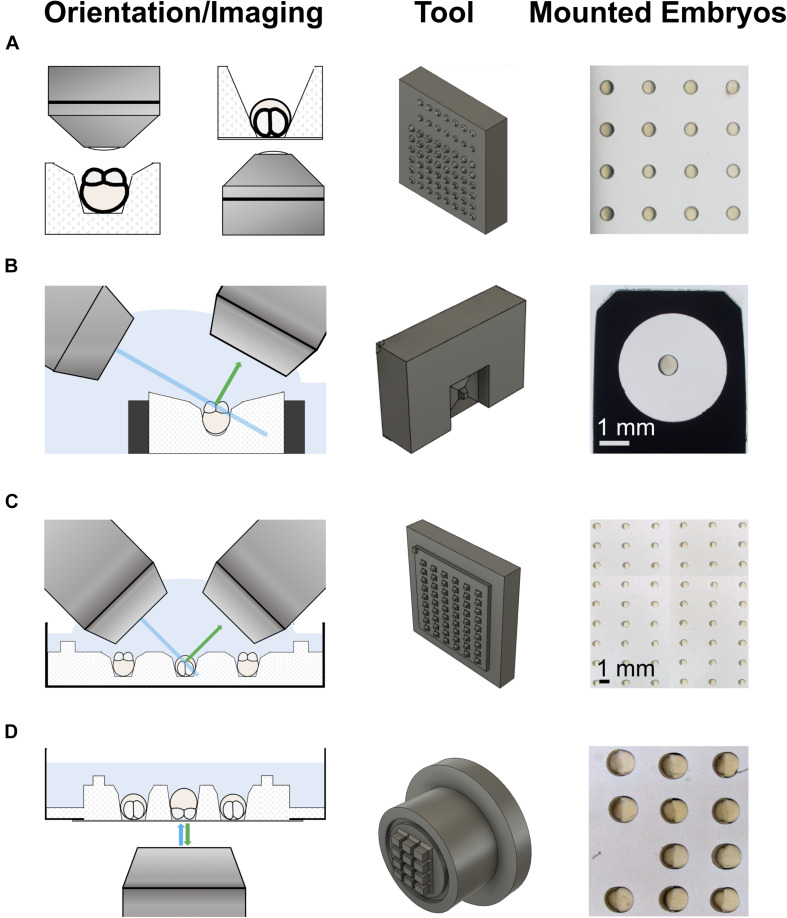
3d-printed tools for imaging on upright and inverted microscopes. The left column shows schematics of the different imaging modalities. The middle column shows the 3d-printed mounting tools. The right column shows examples of embryos mounted using the tools. **(A)** An iterative design strategy was used to optimize pins on 3d-printed mounting tools for different imaging modalities on upright (left) and inverted (right) microscopes. **(B)** Tool to mount embryos in a holder used for imaging on a lattice light-sheet microscope (LLSM). **(C)** Tool to mount embryos in a Petri dish for imaging on an upright microscope, such as a diSPIM. The pins sit on a plate with a slight offset from the actual tool block. This generates a shallow pool on the imaging plate after removal of the medium, allowing direct access for water dipping lenses to image the mounted embryos. **(D)** Tool to mount embryos in a glass-bottom dish for imaging on an inverted microscope, e.g., by spinning disk microscopy. Similar to the tool for Petri dishes, this tool has a feature to produce shallow wells for mounting, a trench that runs around the wells.

We optimized two principal designs for the pins that generate wells in agarose: (i) tapered boxes with a trapezoid cross section and (ii) cone-shaped designs (Supplementary Figures 1–3). We used an iterative design strategy for the pins of the molds by testing prototype molds carrying many different pin designs, and secondary prototyping molds focusing on the refinement of designs that worked well, to finally arrive at the definitive pin design for our mounting tools (Figure 1A).

To determine the range of well sizes for mounting cleavage stage embryos in a stable orientation, we first queried the literature. To ensure that the mounted embryos develop normally, we used the maximum size reported for a reference embryo, i.e., 900 μm in diameter and 1.1 mm extension along the animal-vegetal (AV) axis. We then used PowerPoint software to model wells of different shapes around the reference embryo to achieve mounting in line with the above criteria.

Based on the modeling, and on previously reported designs, we tested two principal geometries for the pins of our molds: (i) tapered boxes, and (ii) cones (Figure 1A and Supplementary Figures 1–3). To determine the ideal dimensions for the pins, we took an empirical approach via iterative design. For the first-generation prototyping mold pin designs, we varied parameters such as the inclination of the tapered walls, the length of the pins, and the cone diameter or the width of individual boxes (Figure 1A).

We tested the various wells generated by the prototype plate for the mounting of early embryos (< 1 dpf). As might be expected, some designs generated wells that were too wide for stable mounting, while others produced narrow wells that deformed and/or killed the embryos. However, other designs allowed stable mounting of embryos. The best designs were chosen for refinement using secondary prototyping molds. On those molds, the parameters (listed above) were varied at a finer scale from the first prototyping mold pin designs. The pins used for the tools used here are the best designs identified by testing of the secondary prototyping molds.

One cone design, and three different box designs of pins were 3d-printed to make a set of LLSM tools. Supplementary Figure 1 describes dimensions and details of the LLSM tool set. The different LLSM tool designs are meant to accommodate for different microscope set ups and for different stages and views to be imaged, also beyond 1 dpf. We used a cone tool that sits 1.4 mm deep in the box on a 0.1 mm spacer for imaging cleavage stage embryos via animal pole views by LLSM (see Supplementary Figure 1).

Similarly, two cone and four box designs of pins were 3d-printed to make a set of tools for imaging on upright microscopes, details described in Supplementary Figure 2. We commonly use the shallow wells generated by the shorter Design 2 pin design for animal pole view imaging of embryos in array format. Orientation of the embryos is more stable in deep wells generated by Design 1 pins, and this tool can be used if the mounted embryos need to be transported over a longer distance. For lateral views, the largest box, Design 4, is commonly used.

Supplementary Figure 3 describes the details of the tools we developed for the imaging of embryos on inverted microscopes. Different box designs of pins were used for the two tools. Besides in funnel-shaped wells, embryos can also be mounted in an upright orientation, i.e., animal pole facing upward/downward, in narrow boxes. While the tool for lateral view imaging has a feature that generates a shallow pool of medium around the mounted embryos, the wells of the animal/vegetal pole tool are themselves deep enough for removal of excess medium from the side.

It is important that there is sufficient spacing between the wells and the well arrays to allow embryos to be gently pushed into wells from the side, e.g., vegetal half first, such that they slide into a pre-oriented position within the well. Although our designs allow reorientation of the embryos within wells, we recommend limiting this to minor adjustments for the highly fragile early embryonic stages (<1 dpf).

The majority of the digital 3d models for the molds were generated on Autodesk Fusion 360 software, and a few first-generation molds (with box-shaped pins) were designed using SOLIDWORKS 3D CAD software. STL files from the 3d models were used as templates for 3d printing by a commercial service, 3d Creation Lab (Ombersley, United Kingdom). The molds were 3d-printed on either a ProJet^TM^ HD 3000(Plus) printer (3d Systems, Wilsonville, United States) in EX200 material, a hard acrylic plastic, or a Objet260 Connex printer (Stratasys, Rehovot, Israel) in rigid opaque materials of the Vero family, typically in black. Both printing technologies offer sufficiently high definition for printing our tools, with the ProJet producing prints of 750 × 750 × 1600 dpi (xyz) and the Objet260 of 600 × 600 × 1600 dpi (xyz), according to the manufacturers.

## Results

### Imaging at the Level of Individual Germ Granules

**FIGURE 2:**
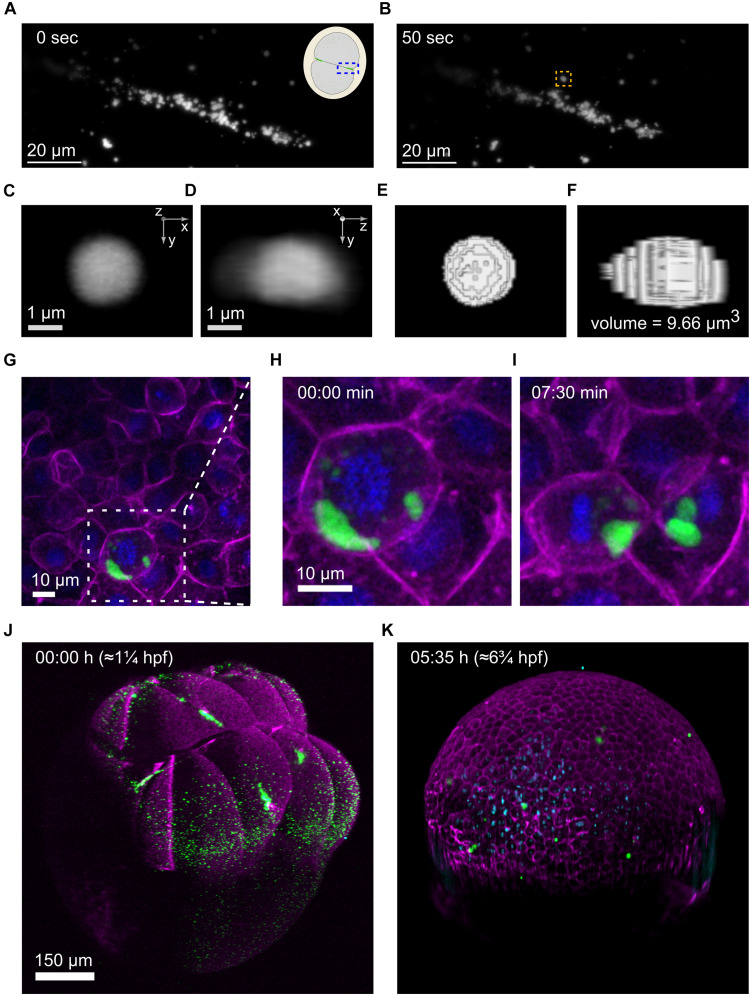
Imaging of germplasm across scales **(A–F)**, Imaging of individual germ granules (RNP complexes). **(A,B)**, Stills from a 4d movie showing germplasm aggregation at the first cleavage furrow imaged on a 3i LLSM at two time points 50 seconds apart. The inset in the top right corner of the earlier time point **(A)** is a schematic illustrating the area that has been imaged in an animal pole view first cleavage embryo, highlighted by a blue box. One specific germ granule is framed in an orange box at the later time point **(B)**. **(C–F)**, This germ granule (at 31 s) was used for volume measurement by threshold segmentation in 3d. **(C,D)**, The granule is shown in two orthogonal views. **(E,F)**, The segmented granule in the same views, together with the measured volume. **(G–I)**, Imaging of individual PGCs. **(G)** Still from a 4d movie of germplasm segregation during cell division of a PGC at the oblong stage, imaged on a spinning disk confocal microscope. Germplasm is labeled by Buc-EGFP (green), membranes by Farnesyl-mCherry (magenta) and chromatin by H2Afv-tagBFP (blue). The middle and right panels show zoom-ins of the area highlighted by a dashed white box in **(G)** at the timepoints in **(H)** and **(I)**. **(J,K)** In toto imaging of germplasm in a zebrafish embryo over a 5.5 h duration on a 3i diSPIM microscope in SPIM mode. The first frame (8-cell, left) and the last frame (gastrula, right) are shown. Four large germplasm masses are observed at the 8-cell stage **(J)** and are partitioned into many PGCs in the gastrula **(K)**. Germplasm is labeled by Buc-EGFP (green), membranes and actin cortex by Farnesyl-mCherry and Utrn-mCherry (magenta), respectively, and chromatin by H2Afv-tagBFP (blue).

The exceptional spatiotemporal resolution offered by lattice light-sheet microscopes makes it an ideal technology to study highly dynamic processes, for instance, the movement of germ granules (Wang et al., 2014). We designed a set of 3D-printed tools that generate wells in LLSM sample holders in the same way that wells are generated using molds with agarose-coated dishes (Figure 1B). The pins of the tools have slightly differing designs, that allow imaging of embryos at various stages and in different orientations, e.g., animal/vegetal pole views versus lateral views. An example of embryos mounted and imaged using our tools is shown in Figures 2A–F, where we acquired 4D movies of germ granules at high speed to determine parameters of germ granule kinetics, e.g., the speed of granule movement in 3D and its volume by thresholded 3d segmentation (Figures 2C–F).

### Imaging at the Cellular Level

For imaging on upright microscopes, the depth of the wells generated by the molds needs to be carefully optimized to facilitate access of water dipping lenses to the embryo, while still holding it in place. Increasing the depth of the wells is beneficial when imaging on inverted microscopes because it reduces turbulence when moving the imaging plate, e.g., during transfer after mounting at a dissection microscope to the imaging microscope (Westhoff et al., 2013); However, this makes it more difficult to orient the embryos within the wells with an eye lash (or other) tool. Another way of preventing turbulence is to cover the mounted sample with a cover slip (Megason, 2009). Alternatively, reducing the medium levels down to a thin layer that covers the embryos in the wells can facilitate the transfer of the imaging plate before the medium is topped up again for imaging (Wuhr et al., 2011). We designed our tools for inverted microscopes according to the latter strategy.

We designed a mold that produces four 3 × 3 arrays of wells for imaging vegetal and animal pole views of embryos on glass-bottom dishes commonly used with inverted microscopes (Supplementary Figure 3B), and a mold that produces a 3 × 4 array of wells for imaging side views (Figure 1D and Supplementary Figure 3A). In Figures 2G–I we show an example where we used the dishes to image germplasm (green), together with membranes (magenta) and chromatin (blue) and examined germplasm segregation during PGC cell divisions. These studies revealed slicing of germplasm masses during cell divisions at mid blastula- early gastrula stages (Figures 2H,I).

### Imaging at the Whole Organism Level

Understanding how early defects in accumulation and segregation of germplasm affect early germline development requires imaging of the entire process, i.e., *in toto* imaging (Megason, 2009). Light-sheet microscopy, also called selective plane illumination microscopy (SPIM), is widely used for such studies (Shah et al., 2019). Selective plane illumination microscopy microscopes are generally upright microscopes, and some have sample holders for Petri dishes whereas others use capillary tubes within which embryos can be mounted. Using similar designs for pins as the ones used for the LLSM tools, we designed molds that generate wells in agarose-coated Petri dishes that can be used to image <1 dpf embryos on a 3i diSPIM microscope (Figure 1C). An example of an embryo before and after 5.5 h of imaging in three channels in 2.5 min intervals is shown in Figures 2J,K. The initial four germplasm masses (Figure 2J) have turned into multiple smaller clusters by fragmentation (Figure 2K).

## Methods

### Reporter Labeling

We used a combination of transgenic and transient reporter expression to label germplasm together with cellular structures. The *Tg(buc:buc-egfp)* transgenic line carries a BAC construct that contains the endogenous *bucky ball* (*buc*) locus with EGFP fused to the *buc* ORF (Riemer et al., 2015). Hence, the expression of the Buc-EGFP fusion protein, which localizes to germplasm, recapitulates endogenous Buc expression. A *Tg(actb2:mCherry- Hsa.UTRN)* transgenic line was used to label the f-actin cortex via the binding of the actin binding domain of Utrophin fused to mCherry (Compagnon et al., 2014).

Synthetic mRNA produced by *in vitro* transcription of *Not*I linearized pCS2 + mCherry-CAAX-*nanos1*-3’UTR reporter construct with the mMessage mMachine SP6 kit was used for the labeling of membranes. The CAAX sequence confers membrane localization via farnesylation, and the *nanos1*-3’UTR gradually directs expression of the reporter into PGCs over the first day of development (Köprunner et al., 2001). Synthetic mRNA produced by *in vitro* transcription of *Not*I linearized pCS2 + H2Afv-tagBFP-*nanos1*-3’UTR reporter construct with the mMessage mMachine SP6 kit was used for the labeling of chromatin by the histone-tagBFP fusion protein (Compagnon et al., 2014).

### Imaging of Germplasm at the Level of Individual Germ Granules (LLSM)

A sample holder for imaging was produced by pipetting 50 μl of molten 1.5% agarose gel into the opening at the end of the LLSM holder. The LLSM tool (Figure 1B) was put over the end of the LLSM holder containing the melted agarose. Once the agarose gel had solidified, 4–5 min, the LLSM tool (mold) was lifted off. This left behind a single well in the center of the agarose within the sample holder.

Buc-EGFP expressing embryos were obtained by crossing female *Tg(buc:buc-egfp)* transgenics with TU wt males. A few embryos were dechorionated manually using tweezers. One embryo was pipetted into the well on the agarose-coated LLSM sample holder using a glass Pasteur pipette and oriented animal pole facing upward using an eye-lash tool. The LLSM sample holder with the embryo was transferred into the medium bath (28°C) on the stage of a LLSM equipped with 0.71NA LWD WI (excitation) and 1.1NA WI (imaging) objectives.

4D images were captured on a 3i Lattice LightSheet microscope with Bessel Beam Illumination of 50 beams with an inner and outer numerical aperture of 0.493 and 0.550, respectively (Intelligent Imaging Innovations, Denver, CO) equipped with a Hamamatsu ORCA-Flash 4.0 sCMOS camera (Hamamatsu Photonics, Skokie, IL) run by SlideBook software (Intelligent Imaging Innovations, Denver, CO). A volume of 53 × 53 × 49.27 μm in 130 slices was acquired in 1.18 s intervals at 62.5x magnification in the green channel (488 nm laser, 5 ms exposure at 10%). The z step size was 0.7 μm, which with a sheet angle of 32.8° gives a voxel volume of 104 × 104 × 379 nm (xyz) after deskew.

The volumes were de-skewed using the SlideBook software. Maximum intensity projections and adjustments of brightness and contrast were done in Fiji software (Schindelin et al., 2012). The 3d volume measurement of an individual germ granule was also carried out in Fiji using the “3d Objects Counter” for the measurement and the “ClearVolume” plugin (Royer et al., 2015) to display 3d projections of the unsegmented and segmented granules.

### Imaging at the Cellular Level (SDM)

A sample holder for imaging was produced by filling a glass-bottom dish with 1 ml of molten 1.7% agarose and placing the tool for lateral view imaging on inverted microscopes (Figure 1D) onto the cover slip at the bottom of the dish. Removal of the tool after solidification of the agarose left behind a 4 × 3 array of wells.

Buc-EGFP expressing embryos were obtained by crossing female *Tg(buc:buc-egfp)* transgenics with TU wt males. To label membranes and chromatin the embryos were injected with 100 pg *mCherry-CAAX-nanos1-3’UTR* and 120 pg *H2Afv-tagBFP-nanos1-3’UTR* synthetic mRNA. After the injection the embryos were dechorionated by treatment for 2 min with 2 mg/ml Pronase (P5147, Merck Life Science UK Limited, Gillingham, United Kingdom). Around high stage the embryos were pipetted into the wells of the sample holder for mounting. Since they readily rested on their sides, they only required minimal reorientation with an eye lash tool.

4D images of three embryos were captured by multipoint acquisition on a Andor Revolution spinning disk confocal microscope (Andor an Oxford Instruments company, Belfast, United Kingdom) equipped with a Andor iXon 897 camera run by Andor iQ software. A volume of 102.4 × 102.4 × 150 μm was acquired in 1.5 min intervals at 40x magnification in the red (561 laser at 20%), green (488 nm laser at 20%) and blue channels (405 nm at 20%). The 40x objective was an oil immersion objective and the step size was 4.84 μm.

Image processing was carried out using Fiji. Maximum intensity projections were generated of five consecutive slices. Medium filter was applied followed by brightness and contrast adjustments.

### Imaging at the Whole Organism Level (SPIM)

The sample holder for the imaging on upright microscopes was produced by filling a Petri dish with 1.5% agarose using the respective tool to cast wells into the solidified agarose. The embryos for imaging were from incrosses of *Tg(buc:buc-egfp)*; *Tg(actb2:mCherry- Hsa.UTRN)* fish and at the 1-cell stage had been injected with 100 pg *mCherry-CAAX-nanos1-3’UTR* and 100 pg *H2Afv-tagBFP-nanos1-3’UTR* synthetic mRNA. At the 4-cell stage the embryos were mounted animal pole facing upward and transferred under a dual inverted selective plane illumination microscope (diSPIM) equipped with two 0.3 NA water dipping 10x objectives (Nikon UK, United Kingdom).

4D images were captured on a 3i Marianas diSPIM (Intelligent Imaging Innovations, Denver, CO) equipped with a Hamamatsu ORCA-Flash 4.0 sCMOS camera (Hamamatsu Photonics, Skokie, IL) run by SlideBook software (Intelligent Imaging Innovations, Denver, CO). A volume of 998.4 × 998.4 × 1136.2 μm was acquired in 142.6 s intervals at 10× magnification in the green (488 nm laser), red (561 nm), and blue (405 nm) channels with an exposure time of 50 ms for each channel. The z step size was 4.234 μm which with a sheet angle of 45° gives a voxel volume of 650 × 650 × 2990 nm (xyz) after deskew.

Image processing comprising of deskewing, cropping, gaussian filter application was carried out in SlideBook software. 3D rendering and brightness/contrast adjustments were carried out in Imaris (Bitplane AG, Zurich, Switzerland).

## Discussion

We developed 3d-printed tools for the mounting of <1 dpf zebrafish embryos on a variety of microscopes. The molds that we have designed carry pins that are negatives of the wells they cast on an agarose-coated sample holder. The dimensions and geometries of the pins have been optimized via iterative design to facilitate positioning of the embryos in a stable orientation whilst still allowing normal development.

Our tool for the LLSM holder facilitates imaging of 1 <dpf embryos on a 3i LLSM with direct access of the water dipping lenses to the embryo, without the use of LMA. To our knowledge, this method is the only one that achieves that for <1 dpf embryos due to the embryos being mounted in the sample holder as opposed to on it. This allows transfer of fragile <1 dpf embryos without the use of LMA because the embryos remain covered by a shallow pool of medium during transfer. This pool is generated by shallow (100–300 μm depth) tapered box that has the pin for the well in its center (Figure 1B and Supplementary Figure 1).

The other tools presented here also carry features that confer compatibility of using shallow wells with transfer of sample holders between microscopes and stable orientation of the mounted embryos. The shallow wells enable easy reorientation of the embryos in the wells, which is essential for efficient mounting of fragile 1 < dpf zebrafish embryos in array format (see Figure 1C). Another advantage is that they give direct access for the imaging objectives to the embryo, which ensures optimal imaging quality.

For the upright imaging tool, the key feature is a box carrying the pins which protrudes 800 μm from the 3d-printed block. This creates an 800 μm high wall surrounding the wells on the agarose-coated well plates. When the medium is removed from the rest of the plate the wall traps medium in a shallow pool above the wells. For the tool for lateral view imaging on inverted microscopes the corresponding feature is a 0.4 μm deep trench running around the surface that carries the pins. This trench fills with agarose during generation of the imaging glass-bottom dish, resulting in agarose walls to generate a pool.

The tools are designed to enable fast and efficient mounting and imaging of <1 dpf embryos to obtain sample sizes compatible with statistical inference in one imaging experiment. In practice, this often means aiming at imaging as many embryos as possible with one imaging holder, without exceeding meaningful time intervals between embryos, and frames in time-lapse experiments. Hence, ideally the wells would be packed as close as possible to one another, to limit time loss due to stage movements between embryos. However, as described in the “Design of Tools” section, space between wells enables easier and better mounting. We found that an arrangement of tight 3 × 3 arrays with enough space to place embryos between them, like the imaging holders produced by our tool for animal/vegetal pole view imaging on inverted microscopes (Supplementary Figure 3), are ideal for that purpose. This also facilitates the imaging of different groups, mutants versus controls, on one holder.

For relatively small sample holders, such as glass-bottom dishes and LLSM holder, size constraints limit the number of wells that can be produced on them. This is less of a problem when using standard Petri dishes (9 cm diameter) as sample holders, but the area that can be imaged is still constrained by the limits in the xy-movement of the microscope’s stage. Our LLSM tools only produce one well, although there would be enough space for at least a 2 × 2 array of wells. We never attempted to produce 2 × 2 or larger arrays of wells in LLSM holders. The reason for that is that we use the LLSM to image highly dynamic processes, such as the movement of germ granules, in the highest possible spatiotemporal resolution. Nevertheless, it should theoretically be possible to mount more than one embryo in LLSM holders using our strategy.

Mounting without the use of viscous media, such as our method, comes with a slight decrease in the stability of oriented embryos. While most of the embryos for lateral view imaging stay in the correct orientation (see Figures 1C,D) a proportion of embryos, 8–25% (average around 14%), for imaging in an upright position may be disturbed from the desired orientation during transfer. The use of mounting media such as LMA or methylcellulose in conjunction with our tools can potentially overcome this issue. However, this is likely to lead to loss of optimal imaging quality due to materials other than the imaging medium being inserted between embryo and objective. The ease of mounting embryos in array formats using our method allows imaging of sample sizes compatible with statistical inference in one experiment. In most cases the advantages and ease of utility of our method outweigh the limitations.

## Data Availability Statement

The raw data supporting the conclusions of this article will be made available by the authors upon request.

## Ethics Statement

The animal study was reviewed and approved by the AWERB, University of Warwick.

## Author Contributions

AZ performed the experiments and prepared the figures and manuscript draft. CAM and HLEC assisted with the lattice light sheet and spinning disk confocal imaging of germ plasm. KS prepared the manuscript draft. All authors contributed to the article and approved the submitted version.

## Conflict of Interest

The authors declare that the research was conducted in the absence of any commercial or financial relationships that could be construed as a potential conflict of interest.

## Publisher’s Note

All claims expressed in this article are solely those of the authors and do not necessarily represent those of their affiliated organizations, or those of the publisher, the editors and the reviewers. Any product that may be evaluated in this article, or claim that may be made by its manufacturer, is not guaranteed or endorsed by the publisher.
