# Supplementary material for: Tools to Image Germplasm Dynamics During Early Zebrafish Development
Source: Front Cell Dev Biol. 2021 Aug 13;9:712503. doi: 10.3389/fcell.2021.712503 (PMC8414583; doi:10.3389/fcell.2021.712503)
Supplement: Supplementary file 1 [file Data_Sheet_1.PDF]

Figure S1: The Tool for LLSM imaging.

(A), Schematic representation of a 3d model of LLSM tools, with details of components and features (B-E). The pins are either cones for animal/vegetal pole imaging (B) or tapered boxes for lateral view imaging (C). Dimensions are given in millimeters (mm). The LLSM tools are connected by 5 mm rods for printing in one piece. Individual tools can be broken or cut off from the connectors. The pins (B-C) sit on the bottom of 1.3 or 1.4 mm deep boxes (D) cut out of a 4 mm thick block. The pins may either sit directly on the bottom of the box or may be elevated by spacers of three different heights (E).

Figure S2: Tool for imaging germplasm across scales using an upright microscope.

(A), Schematic of 3d model for the upright microscope tools. (B,C), Details of features and components, with dimensions in millimeters. (B), Tools with box-shaped pins for lateral view imaging work best with the largest Design 4 boxes. (C), Cone-shaped pins for animal/vegetal pole view imaging in array format using a shallow Design 2 pin tool. Orientation of the embryos is more stable in deep wells generated by Design 1 pins, which can be used if mounted embryos need to be transported over a distance.

Figure S3: Tool for imaging of germplasm across scales on an inverted microscope.

(A,B) Schematic of 3d models of tools used with inverted microscopes, with details of features and components, and dimensions in millimeters. Here, tapered box designs are used for both lateral (A) and animal/vegetal pole view (B) imaging tools. Wells generated by the animal/vegetal pole view imaging tool (B) are deep enough for removal of liquid from the side after embryo mounting; An additional feature for generation of a pool of medium surrounding the mounted embryo allows orientation and stable transfer of mounted embryos to the imaging microscope.
