# Supplementary figures and images for: Tools to Image Germplasm Dynamics During Early Zebrafish Development
Source: Front Cell Dev Biol. 2021 Aug 13;9:712503. doi: 10.3389/fcell.2021.712503 (PMC8414583; doi:10.3389/fcell.2021.712503)

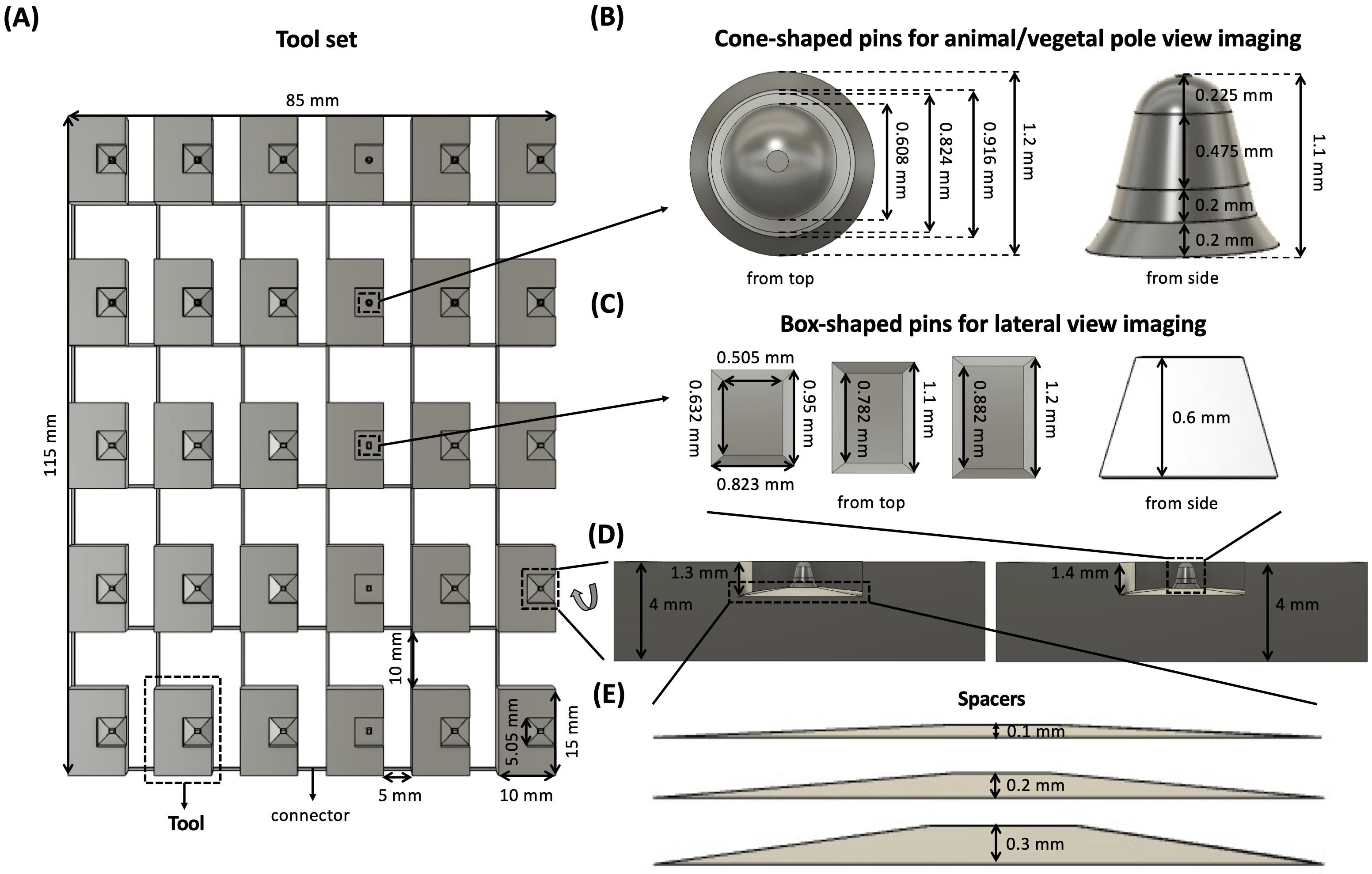

Supplement: Supplementary file 2 [file Image_1.PNG]

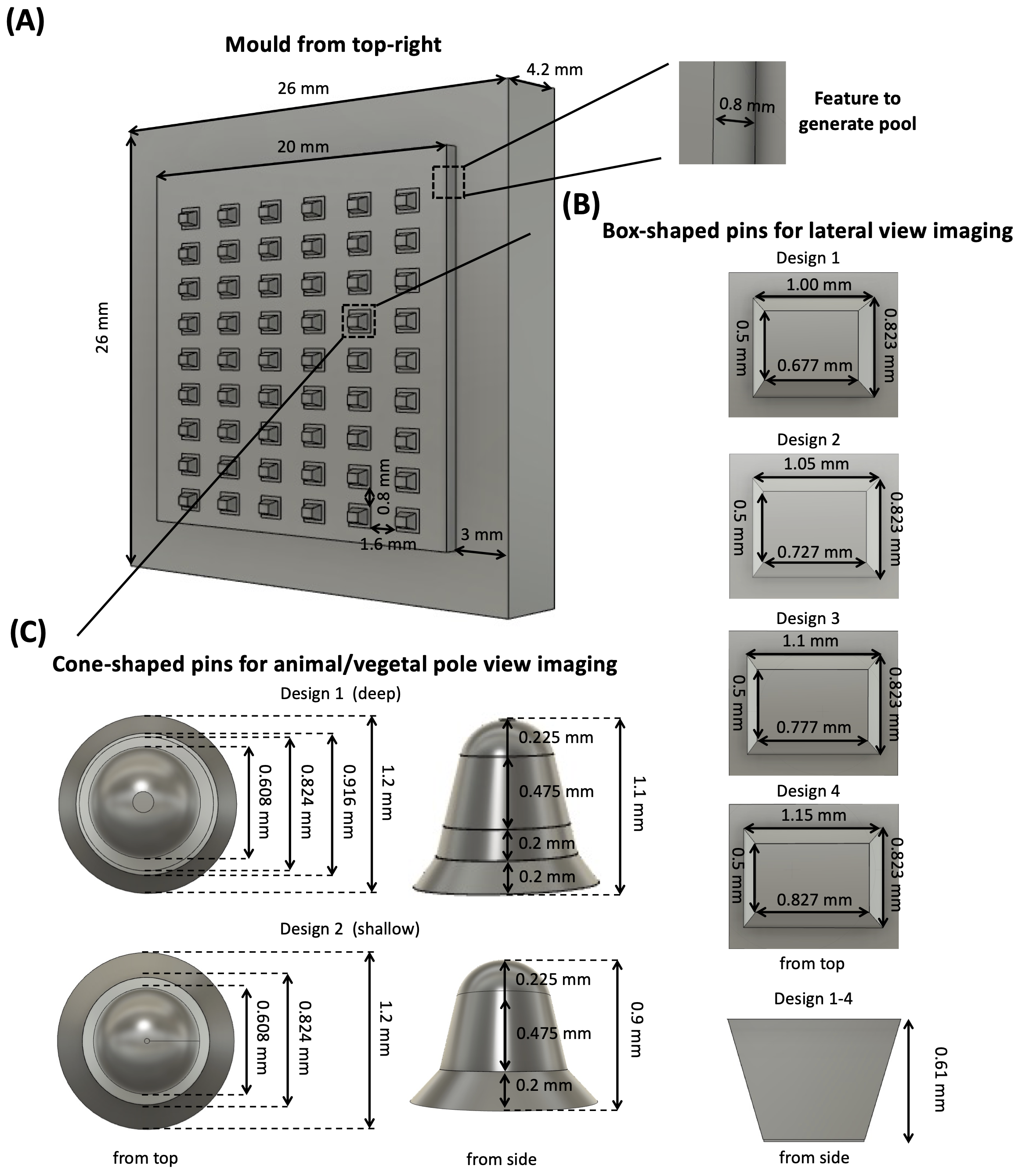

Supplement: Supplementary file 3 [file Image_2.PNG]

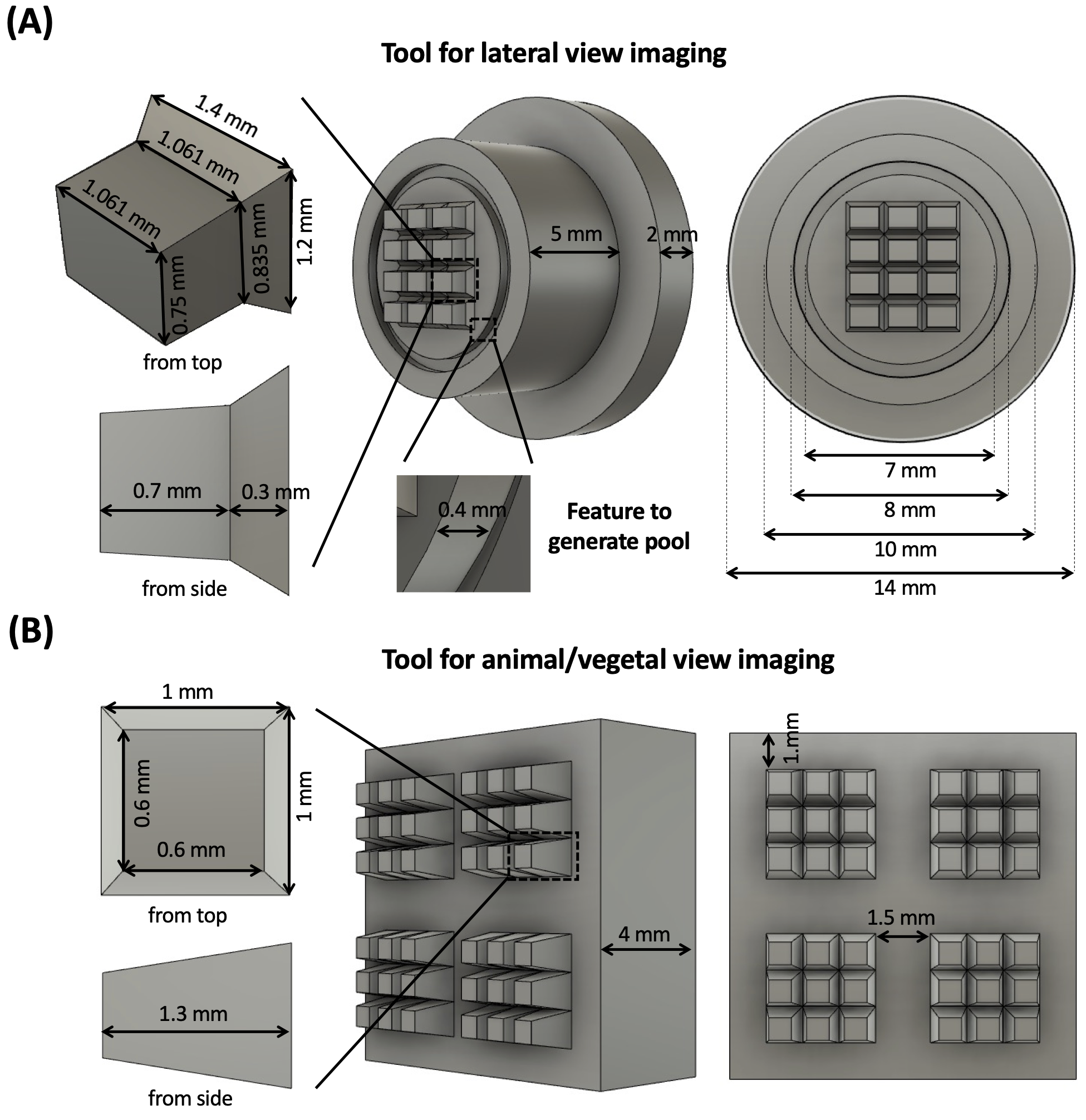

Supplement: Supplementary file 4 [file Image_3.PNG]
